# Rapid regulation of microRNA following induction of long-term potentiation *in vivo*

**DOI:** 10.3389/fnmol.2014.00098

**Published:** 2014-12-09

**Authors:** Greig Joilin, Diane Guévremont, Brigid Ryan, Charles Claudianos, Alexandre S. Cristino, Wickliffe C. Abraham, Joanna M. Williams

**Affiliations:** ^1^Brain Health Research Centre, University of OtagoDunedin, New Zealand; ^2^Department of Anatomy, Otago School of Medical Sciences, University of OtagoDunedin, New Zealand; ^3^Queensland Brain Institute, The University of QueenslandBrisbane, QLD, Australia; ^4^Department of Psychology, University of OtagoDunedin, New Zealand

**Keywords:** long-term potentiation, microRNA, maintenance, synaptic plasticity, memory

## Abstract

Coordinated regulation of gene expression is essential for consolidation of the memory mechanism, long-term potentiation (LTP). Triggering of LTP by *N*-methyl-D-aspartate receptor (NMDAR) activation rapidly activates constitutive and inducible transcription factors, which promote expression of genes responsible for LTP maintenance. As microRNA (miRNA) coordinate expression of genes related through seed sites, we hypothesize that miRNA contribute to the regulation of the LTP-induced gene response. MiRNA function primarily as negative regulators of gene expression. As LTP induction promotes a generalized rapid up-regulation of gene expression, we predicted a complementary rapid down-regulation of miRNA levels. Accordingly, we carried out global miRNA expression profiling in the rat dentate gyrus 20 min post-LTP induction *in vivo*. Consistent with our hypothesis, we found a large number of differentially expressed miRNA, the majority down-regulated. Detailed analysis of miR-34a-5p and miR-132-3p revealed this down-regulation was transient and NMDAR-dependent, whereby block of NMDARs released an activity-associated inhibitory mechanism. Furthermore, down-regulation of mature miR-34a-5p and miR-132-3p occurred solely by post-transcriptional mechanisms, occurring despite an associated up-regulation of the pri-miR-132 transcript. To understand how down-regulation of miR-34a-5p and miR-132-3p intersects with the molecular events occurring following LTP, we used bioinformatics to identify potential targets. Previously validated targets included the key LTP-regulated genes *Arc* and glutamate receptor subunits. Predicted targets included the LTP-linked kinase, *Mapk1*, and neuropil-associated transcripts *Hn1* and *Klhl11*, which were validated using luciferase reporter assays. Furthermore, we found that the level of p42-Mapk1, the protein encoded by the *Mapk1* transcript, was up-regulated following LTP. Together, these data support the interpretation that miRNA, in particular miR-34a-5p and miR-132-3p, make a surprisingly rapid contribution to synaptic plasticity via dis-inhibition of translation of key plasticity-related molecules.

## INTRODUCTION

Long-term potentiation (LTP) exhibits many properties key to a mnemonic device. Paramount among these is its remarkable persistence (Abraham et al., 2002). While induction of LTP is dependent largely on activation and trafficking of 2-amino-3-(3-hydroxy-5-methyl-isoxazol-4-yl) propanoic acid (AMPA) and *N*-methyl-D-aspartate (NMDA)-subtypes of glutamate receptors, its persistence is dependent on regulated protein synthesis at synapses and activation of both constitutive (e.g., cAMP response element-binding protein; CREB) and inducible (e.g., early growth factor-1; Egr1) transcription factors (Abraham and Williams, 2003). The breadth of LTP-associated gene expression in the dentate gyrus has been highlighted by our genome-wide analyses. We have shown that perforant path LTP in awake rats dramatically up-regulates transcription at 20 min and 5 h post-LTP induction, followed by a generalized down-regulation at 24 h (Ryan et al., 2011, 2012). Our bioinformatics analysis showed that these regulated genes form highly significant networks, comprising groups of co-regulated genes and “hub” molecules that are likely to act as central controllers of the LTP-related gene response. Consistent with the dogma of the field, the networks engaged 20 min post-LTP induction featured transcription factors as central hubs and predicted that these rapidly responding gene networks contribute to the regulation of protein kinase activity and calcium dynamics. Interestingly, the analysis also predicted important roles for microRNA (miRNA) in the control of LTP-related gene expression, even at this early time-point.

Mature miRNA are generally considered as negative regulators of gene expression (Fabian et al., 2010). The primary (pri-miRNA) transcript contains multiple double-stranded miRNA stem-loops, and precursor transcript (pre-miRNA). Mature single-stranded miRNA are liberated following sequential cleavage of the pri-miRNA by the RNase III enzymes Drosha and Dicer. The mature miRNA initiates translational arrest or mRNA degradation through either partial or complete complementation with its target mRNA. Importantly, partial complementation allows for one miRNA to regulate the translation of a large number of target mRNA. Thus, although induction of regulated transcription is thought to be the primary driver of LTP-related gene expression, we hypothesized that miRNA may play central roles in regulating translation of pre-existing and newly produced transcripts. Indeed, we have reported decreases in miR-24-3p and miR-34a-5p expression at 5 h post-LTP in awake adult rats (Ryan et al., 2012), and others have shown regulation of specific miRNA both in anaesthetized rats (Wibrand et al., 2010, 2012) and *in vitro* (Park and Tang, 2009; Lee et al., 2012). Given our observation of overall increases in gene expression in extracts from whole dentate gyri prepared at 20 min and 5 h, and the enhanced synaptic protein synthesis previously associated with the maintenance of LTP (Krug et al., 1984; Otani et al., 1989; Schuman et al., 2006), we hypothesized that perforant path LTP induced in awake rats would be associated with a rapid down-regulation of specific miRNA, representing a novel mechanism for regulating memory-related gene expression.

## MATERIALS AND METHODS

### ELECTROPHYSIOLOGY

Long-term potentiation was induced in awake freely moving adult male Sprague–Dawley rats (4–5 months). Stimulating and extracellular recording electrodes were chronically implanted bilaterally into the perforant pathway and dentate gyrus hilus, respectively, under ketamine (75 mg/kg, s.c.) and Domitor (0.5 mg/kg, s.c.) anesthesia (Williams et al., 2007). LTP was induced unilaterally using 50 trains (50T) of brief delta-burst high-frequency stimulation (DBS, 400 Hz), known to produce a persistent form of LTP (Abraham et al., 2002; Bowden et al., 2012). Baseline-only stimulation in the contralateral hemisphere was used as a within-animal control. In some experiments (*n* = 4), the NMDAR antagonist (RS)-3-(2-carboxypiperazin-4-yl)-propyl-1-phosphonic acid (CPP; Tocris) was used (7.5 mg/kg, i.p.), followed 90 min later by the same DBS. At appropriate times following DBS (20 min: *n* = 10; 5 h: *n* = 5–10; 24 h: *n* = 5), animals were anesthetized with isoflurane, decapitated, and the matched control and stimulated dentate gyri dissected and stored at -80°C. All animal manipulations were approved by the University of Otago Animal Ethics Committee.

### RNA EXTRACTION

Total RNA, including miRNA, was isolated from individual control and stimulated dentate gyri using Trizol (Invitrogen) and total RNA purification columns (Norgen Biotek), as per our established protocols (Ryan et al., 2011, 2012).

### AFFYMETRIX microRNA ARRAYS

RNA (1 μg) was labeled (FlashTag; Genisphere) and hybridized to Affymetrix GeneChip miRNA arrays (*n* = 4) at the Adelaide Microarray Centre. Data collected were analyzed by miRNA QCTool 1.0.33.0 (Affymetrix) using the recommended workflow. miRNA were identified as being differentially expressed using dual selection criteria (two-tailed Student’s *t*-test *p* < 0.05; fold change ± 0.15; Ryan et al., 2011, 2012). Data for differentially expressed homologous miRNA were grouped together and averaged.

### REVERSE TRANSCRIPTION QUANTITATIVE PCR (RT-qPCR)

*Mature miRNA expression* was measured using the TaqMan miRNA RT-qPCR system (Applied Biosystems) and primers based on miRbase 17. MiRNA-specific cDNA (0.04 ng/μl) was made from sample RNA. RT-qPCR was performed in triplicate and expression normalized to Y1 RNA. *Primary and precursor miRNA expression* was measured using a generic conversion of cDNA to RNA (25 ng/μl; High Capacity RNA to cDNA kit; Applied Biosystems). Primary transcripts were profiled using the TaqMan pri-miRNA RT-qPCR system, while the precursors were profiled using miScript pre-miRNA primers (Qiagen) with SYBR Green I (Roche). The manufacturer’s protocols were used in each instance. The C_q_ values of the pre-miRNA hairpin were >35 and greater than the pri-miRNA values. This is likely due to the competition occurring between closing of the hairpin and annealing of the primers during the amplification process. We concluded that the pre-miRNA levels could not be assessed reliably. Efficiencies of the primary and mature miRNA primers were between 90 and 105% with the *R*^2^ ≥ 0.98. Efficiencies for the pre-miRNA primers were higher or unable to be obtained. The geometric average of triplicate C_q_ values for each sample was normalized to Y1 RNA for mature miRNA and to hypoxanthine phosphoribosyltransferase (HPRT) for pri-miRNA. Using the data for the individual matched control and stimulated dentate gyri, the 2^-ΔΔCp^ method was used to give a fold change with outliers determined using the Grubb’s test (α = 0.05). Significant differential expression between hemispheres was determined by a one sample Student’s *t*-test (*p* < 0.05).

### IDENTIFICATION AND CLASSIFICATION OF mRNA TARGETS

Confirmed targets of these miRNA were investigated using TarBase v6.0 (http://diana.cslab.ece.ntua.gr/DianaToolsNew/index.php?r=tarbase/index), a manually curated database for experimentally validated interactions (Vergoulis et al., 2012). Predicted miRNA targets were identified using two approaches.(1) The miRNA2function tool in the miRNA Body Map program (Mestdagh et al., 2011) integrates all miRNA targets predicted by TargetScanHuman v5.1, MicroCosm Targets v5, miRDB v3, TarBase v.5c, miRecords v2, DIANA-microT v3.0, RNA22 (August 2007), and PITA v6. Targets predicted by at least three algorithms and expressed in rat CA1 neuropil (Cajigas et al., 2012) were selected for further study. (2) The union of predictions made by nine algorithms [DIANA-microT v3.0, doRiNA (PicTar2), miRanda (August 2010), MiTarget2 (miRDB v4), miRWalk (March 2011), PITA v6, RNA22 (May 2008), RNAhybrid v2.1, TargetScanS v6.2] were filtered using the LTP KEGG list, with those predicted by at least four algorithms with sites in the rat genome selected for further study.

### LUCIFERASE ASSAYS

Luciferase assays were performed as previously described (Cristino et al., 2014). In brief, annealed double stranded oligomers including the predicted rno-miR-132-3p miRNA binding elements (MREs) within the 3′ UTR of *Gria2, Hn1, Klhl11,* and *Mapk1* transcripts, as well as mutant sequences where all guanines and cytosines were changed to adenines, and the perfect complimentary sequence to rno-miR-132-3p (**Table 1**), were cloned into the ψCheck-2 vector (Promega) and digested with XhoI and NotI-HF. Recombinant vectors were sequenced (Australian Genome Research Facility; Brisbane Node) to confirm sequence validity. COS-7 cells, seeded on 24-well plates (5 × 10^4^ cells/well; 4 h), were co-transfected with recombinant plasmid (400 ng/well), and either miRIDIAN miR-132-3p mimic or cel-miR-239b negative control sequences (Dharmacon, 240 pmol/well; **Table 1**) using Lipofectamine LTX with Plus Reagent (Invitrogen, 2.5 μl/well; 24 h). Using the Dual-Luciferase Reporter Assay System (Promega), cells were lysed and the lysate either undiluted or diluted (1:2–1:20) to prevent signal saturation during reading of the plate. Firefly and *Renilla* luciferase activities were measured sequentially, according to the manufacturer’s instructions (POLARstar OPTIMA, BMG Labtech). The *Renilla* luciferase expression measured the effect of sequence binding to the insert while the firefly luciferase was used as an internal normaliser for plasmid transfection. All data were normalized to the plasmid-only transfections. Four independent transfection experiments were performed. Significant differences between ratios were determined using a two-tailed unpaired Student’s *t*-test (*p* < 0.05).

**Table 1:** Oligonucleotides including exact match, target and mutated sequences of rno-miR-132-3p MRE that were cloned into the ψCheck-2 vector.

Name of Insert	Sequence
miR-132-3p Perfect Match	CAGTGACTCTCGAGCAGCGACCATGGCTGTAGACTGTTAGACGCGGCCGCCAGTGACT
*Gria2* wildtype NM_017261.2 6745-72	CAGTGACTCTCGAGCAGATGAGGAGCAAGGCAAGGCTGTCAATTGACGCGGCCGCCAGTGACT
*Hn1* wildtype NM_001005876 1289-314	CAGTGACTCTCGAGCAGGTACTTCTTAGTCCTGGACTGTTGCTGACGCGGCCGCCAGTGACT
*Klhl11* wildtype NM_001105838 2232-56	CAGTGACTCTCGAGCAGGGCTGGAGATCCTTGGACTGTTACTGACGCGGCCGCCAGTGACT
*Mapk1* wildtype XM_006248658.1 4971-5000	CAGTGACTCTCGAGCAGACTTACTGTGCTATTGCATGACTGTTAAGGACGCGGCCGCCAGTGACT
*Gria2* mutant	CAGTGACTCTCGAGCAGATGAGGAGCAAGGCAAATATATCAATTGACGCGGCCGCCAGTGACT
*Hn1* mutant	CAGTGACTCTCGAGCAGGTACTTCTTAGTCCTGAAATATTGCTGACGCGGCCGCCAGTGACT
*Klhl11* mutant	CAGTGACTCTCGAGCAGGGCTGGAGATCCTTGAAATATTACTGACGCGGCCGCCAGTGACT
*Mapk1* mutant	CAGTGACTCTCGAGCAGACTTACTGTGCTATTGCATAAATATTAAGGACGCGGCCGCCAGTGACT
rno-miR-132 mimic	UAACAGUCUACAGCCAUGGUCG
cel-miR-239b Negative Control	UUGUACUACACAAAAGUACUG

*Target sequences are underlined*.

### WESTERN BLOT ANALYSIS

Western blot analysis was performed essentially as previously described (Williams et al., 2007). In brief, whole cell protein extracts were prepared using Cell Lysis Buffer (BioVision) and separated by SDS-PAGE (9%) and transferred to a nitrocellulose membrane (Whatman; GE Healthcare). Non-specific binding to membranes was blocked by incubation in Odyssey blocking buffer. Membranes were probed with anti-mouse and anti-rabbit antibodies recognizing p42-Mapk1 and p44-Mapk3 (New England Biolabs) and tubulin (Abcam) respectively. Antibody binding was detected by incubation with appropriate conjugated secondary antibodies and visualized using fluorescent secondary antibodies (anti-mouse IRDye^®^ 680; anti-rabbit IRDye^®^ 800; Licor) and quantified using an infrared scanner (Odyssey^®^ Licor) and the accompanying ImageStudio software. Each sample was normalized to tubulin levels. Fold changes were then calculated between matched control and stimulated dentate gyri, and averaged across groups. Significant differences between ratios were determined using one sample Student’s *t*-test (*p* < 0.05).

## RESULTS

### LTP RAPIDLY REGULATES microRNA EXPRESSION

**FIGURE 1:**
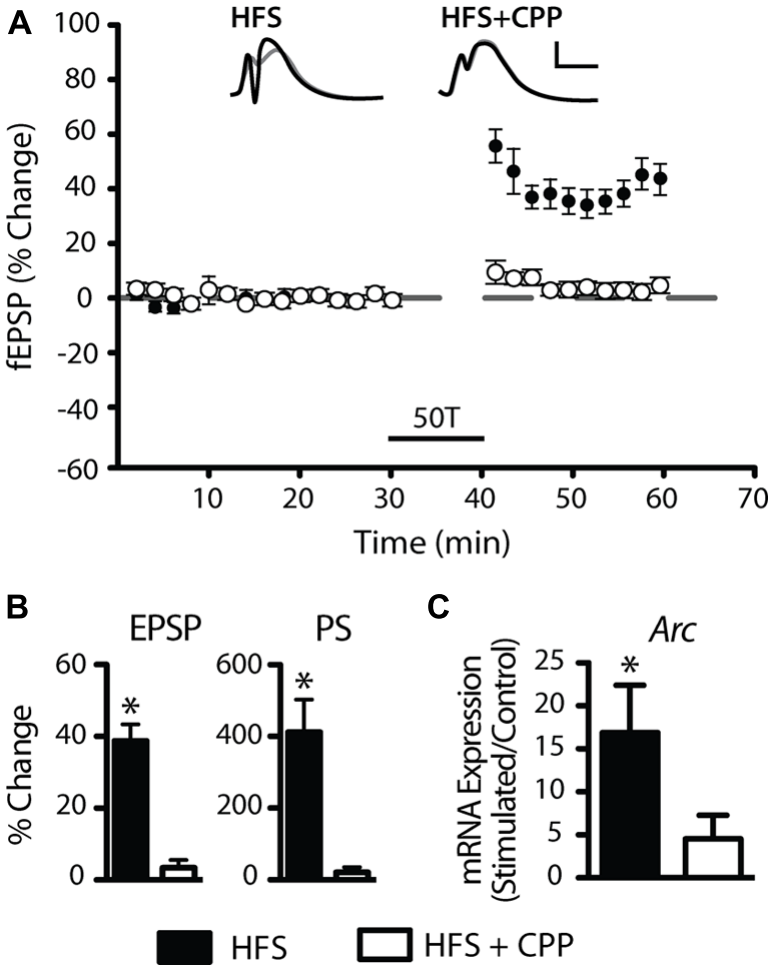
**Induction of robust long-term potentiation (LTP) in the perforant path of awake adult rats. (A,B)** Average (±SEM) field evoked postsynaptic potential (fEPSP) and population spike (PS) responses expressed as percentage of baseline values. At least a 10% increase in the fEPSP and 50% increase in the PS was required for acceptance as LTP induction. Delta-burst high-frequency stimulation (DBS) elicited by 50T resulted in a reliable and robust LTP (filled circles) that was blocked by the *N*-methyl-D-aspartate receptor (NMDAR) antagonist CPP (open circles). Inset waveforms are averages of 10 sweeps taken just before DBS and 15–20 min after. Calibration bars: 5 ms; 5 mV. **(C)** DBS induced a significant increase in *Arc* mRNA levels, a well-characterized LTP-regulated gene that was also blocked by CPP. RT-qPCR was performed in triplicate and *Arc* expression normalized to HPRT. Expression values: average ± SEM; one sample two-tailed *t*-test, *p < 0.05.

Long-term potentiation was induced in freely moving animals using our established DBS protocol (Bowden et al., 2012). DBS resulted in significant increases in the field excitatory postsynaptic potential (fEPSP) and population spike (PS; **Figures 1A,B**) measured 15–20 min post-DBS (*n* = 10), as well as a dramatic up-regulation of activity-related cytoskeletal protein (*Arc*) mRNA, a canonical LTP-associated gene (RT-qPCR: tetanised/control: *p* = 0.02, *n* = 10; one-sample *t*-test; **Figure 1C**). Furthermore, induction of LTP was blocked (**Figure 1B**) and the increase in *Arc* expression was significantly curtailed (*p* = 0.44, *n* = 4; **Figure 1C**) by the NMDAR antagonist, CPP.

**FIGURE 2:**
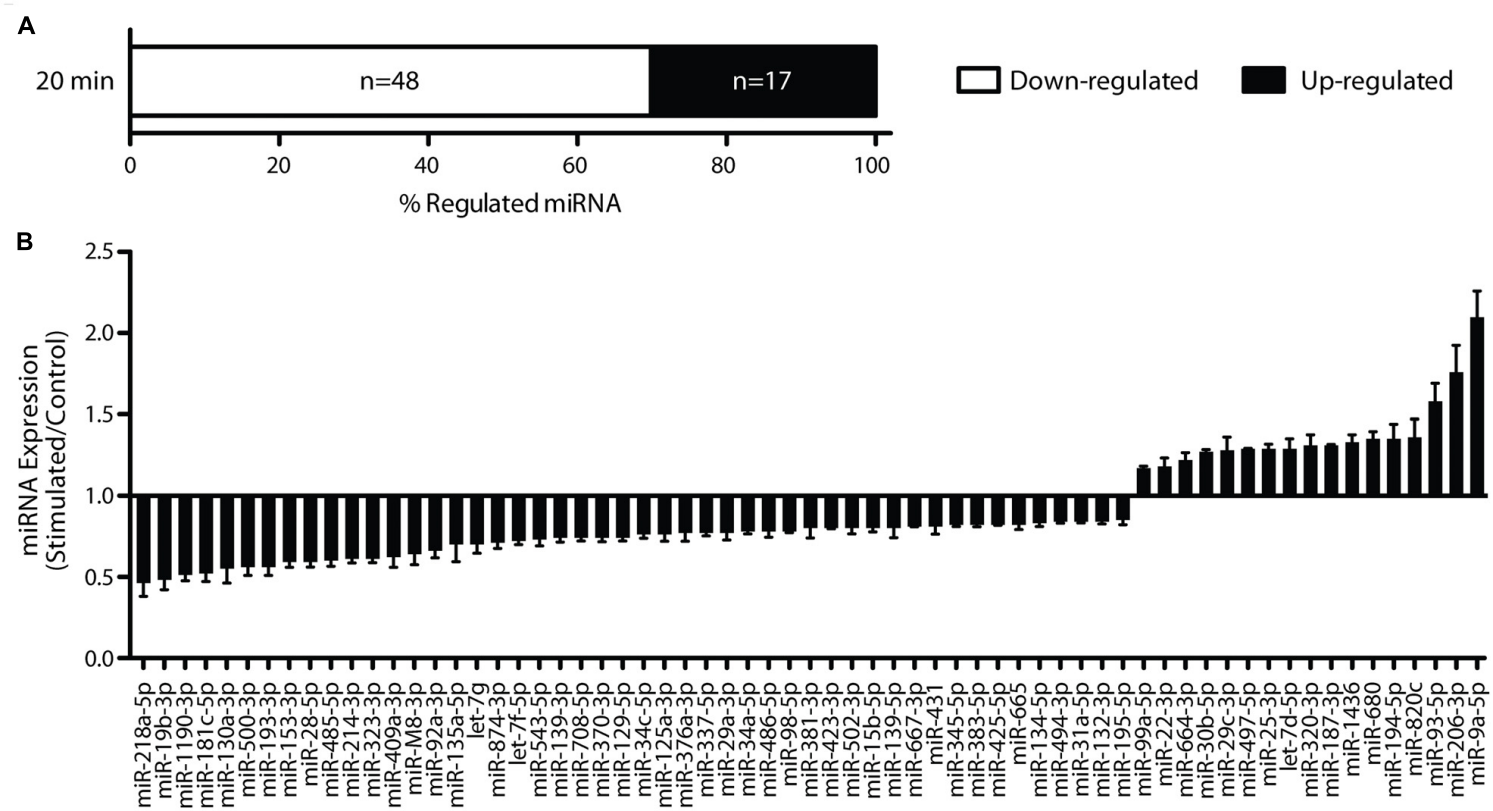
**Long-term potentiation rapidly regulates miRNA 20 min post-LTP. (A)** Rapid regulation of miRNA levels revealed by Affymetrix miRNA array analysis. **(B)** Sixty-five miRNA were found to be differentially expressed using dual selection criteria (two-tailed Student’s *t*-test *p* < 0.05; fold change ± 0.15), with 17 up-regulated and 48 down-regulated.

To test our hypothesis that the rapid up-regulation of LTP-related gene expression is associated with a rapid and generalized down-regulation of miRNA, we carried out miRNA expression profiling of matched LTP-stimulated and control dentate gyri 20 min post-LTP (Affymetrix GeneChip miRNA hybridisation arrays; *n* = 4 pairs). Using dual selection criteria, we identified 65 unique miRNA differentially expressed in response to DBS (**Figure 2**). Consistent with our hypothesis, the majority (74%, 48 miRNA) were down-regulated when compared to their matched contralateral control hemispheres and 26% (17 miRNA) were up-regulated. Most changes were modest in magnitude, with two–thirds showing a fold change less than ±0.40 and only four miRNA (miR-181c-5p, miR-19b-3p, miR-218a-5p, miR-9a-5p) showing more than a twofold change. This degree of regulation is consistent with our previous LTP-related gene expression studies (Ryan et al., 2011, 2012), and with previous studies investigating LTP-related changes in miRNA in anesthetized animals (Wibrand et al., 2010, 2012). However, this is the first study to our knowledge showing rapid down-regulation of a cohort of miRNA in response to LTP induction.

### RAPID DOWN-REGULATION OF MATURE microRNA, miR-34a-5p AND miR-132-3p, IS NMDAR DEPENDENT

**FIGURE 3:**
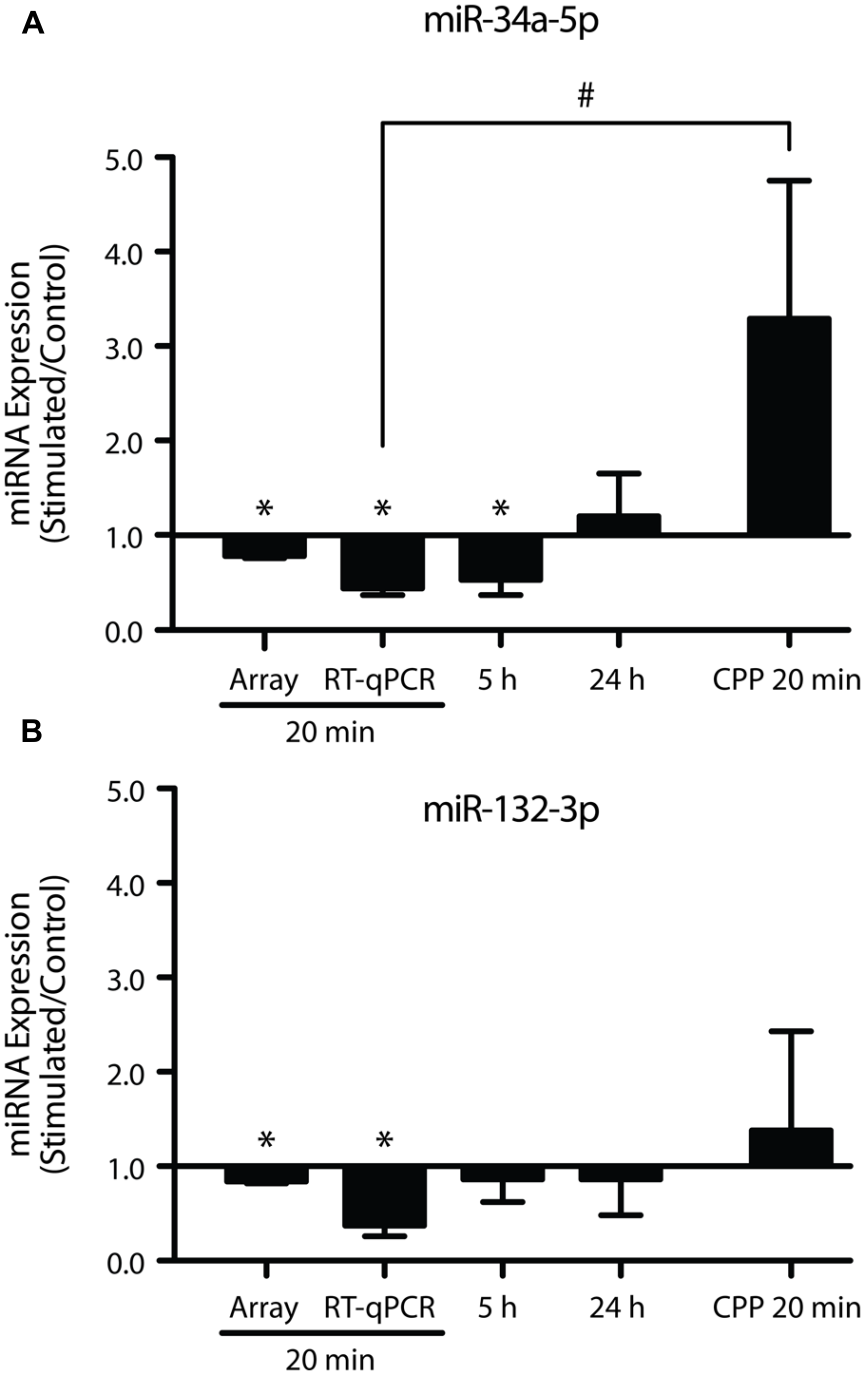
**Long-term potentiation differentially regulates mature miR-34a-5p and miR-132-3p. (A)** Down-regulation of miR-34a-5p was observed 20 min post-LTP, (microarray data validated using RT-qPCR) that persisted for 5 h (Ryan et al., 2012) but returned to baseline by 24 h and was blocked by CPP (20 min; **B)** Down-regulation of miR-132-3p at 20 min (but not 5 or 24 h post-LTP) was blocked by CPP. RT-qPCR was performed in triplicate and expression normalized to Y1 RNA. Expression values: average ± SEM; one sample two-tailed *t*-test, *p < 0.05; independent two-tailed *t*-test, #*p* < 0.05.

A subset of the rapidly down-regulated miRNA (miR-34a-5p, miR-34c-5p, miR-132-3p, miR-181c-5p, miR-214-3p) were chosen for more in-depth analysis by RT-qPCR, based on previous associations with plasticity processes (Wayman et al., 2008; Schonrock et al., 2010; Agostini et al., 2011; Zovoilis et al., 2011; Ryan et al., 2012). Using individual TaqMan qPCR assays, we confirmed reduced expression of miR-34a-5p and miR-132-3p (miR-34a-5p: *p* = 0.0001, *n* = 8; miR-132-3p: *p* = 0.001, *n* = 8; **Figure 3**), but not miR-34c-5p (*p* = 0.14, *n* = 9), miR-181c-5p (*p* = 0.46, *n* = 10) or miR-214-3p (*p* = 0.65, *n* = 9). While previously we have shown that miR-34a-5p is down-regulated at 5 h post-DBS (Ryan et al., 2012), further analyses showed no significant down-regulation of miR-132-3p at this time-point (*p* = 0.58, *n* = 7; **Figure 3B**), thus suggesting that the peak of its reduction lies closer to 20 min. At 24 h post-DBS, a time we previously reported to be associated with a generalized down-regulation of mRNA expression (Ryan et al., 2012), there was no alteration in the expression of either miR-34a-5p (*p* = 0.67, *n* = 4, **Figure 3A**) or miR-132-3p (*p* = 0.74, *n* = 4, **Figure 3B**). It is of note that we found no significant regulation at any time point of miR-212-3p, which is derived from the same primary transcript as miR-132-3p (20 min: *p* = 0.93; 5 h: *p* = 0.63; 24 h: *p* = 0.47; *n* = 4–5). These findings highlight a potential post-transcriptional regulation of miRNA levels in response to LTP induction.

As LTP induction is crucially dependent on activation of NMDARs, we tested whether down-regulation of miR-34a-5p or miR-132-3p was dependent on the activation of NMDARs (Abraham and Mason, 1988). When DBS was given in the presence of the NMDAR antagonist CPP, LTP of both the fEPSP and PS was blocked (**Figures 1B,C**), as was the down-regulation of miR-34a-5p and miR-132-3p. Indeed, CPP in combination with tetanic stimulation led to increased but highly variable levels of miR-34a-5p in the tetanised hemisphere (*p* = 0.22, *n* = 4; **Figure 3**), which when compared to the 20 min LTP-stimulated, non-drug treated group was significantly different (*p* = 0.02). This finding of an evoked activity-related increase in miRNA is in accord with the findings of Wibrand et al. (2010) in urethane-anesthetized animals, and raise the possibility that in awake animals the NMDAR-mediated reduction in the levels of mature miR-34a-5p and miR-132-3p out-competes a second, NMDAR-independent process, working to increase them.

### REGULATION OF PRIMARY TRANSCRIPTS DOES NOT PARALLEL REGULATION OF MATURE miRNA

**FIGURE 4:**
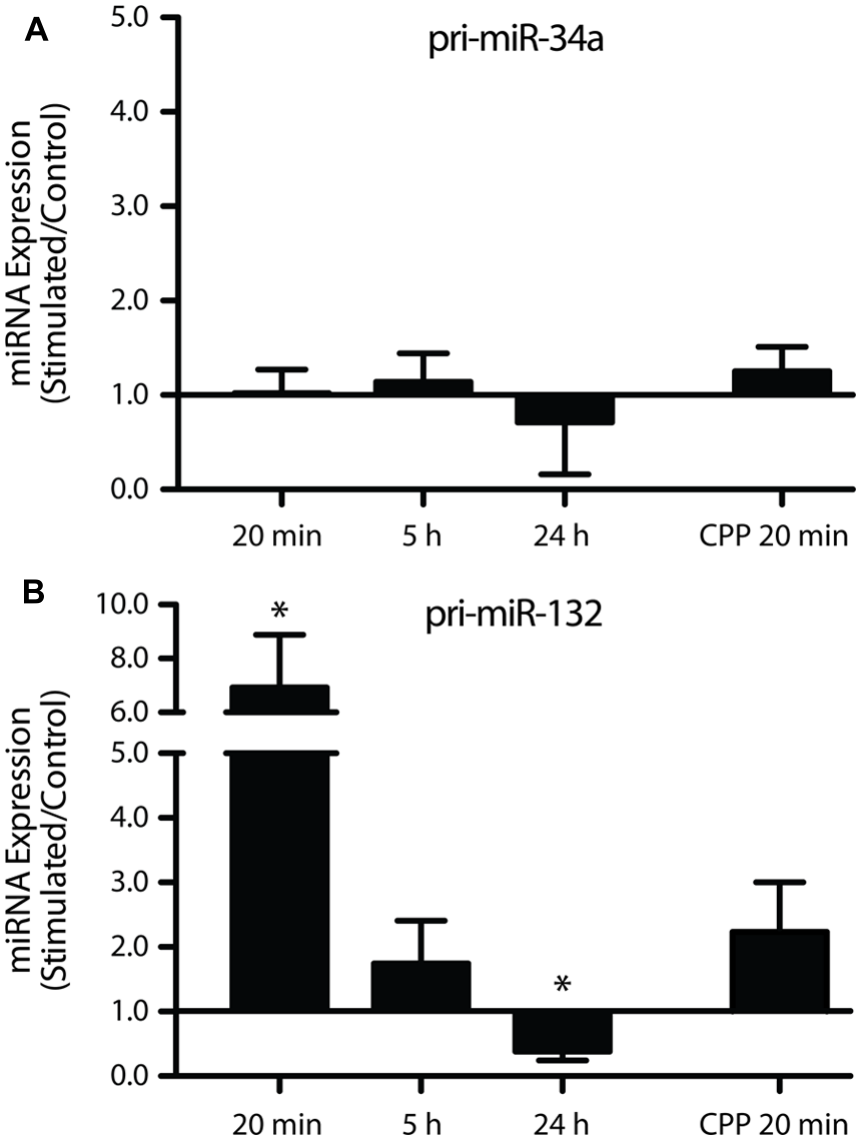
**Long-term potentiation differentially regulates the primary miRNA transcripts of miR-34a-5p and miR-132-3p. (A)** Pri-miR-34a was not differentially expressed 20 min, 5 or 24 h post DBS, or sensitive to CPP. **(B)** miR-132/212 expression was significantly increased at 20 min, unchanged at 5 h and significantly decreased at 24 h. This effect was partially blocked by CPP. Primary transcripts were normalized to HPRT. Expression values: average ± SEM; one sample two-tailed *t*-test *p < 0.05.

One possible mechanism underlying the LTP-induced down-regulation of miR-34a-5p and miR-132-3p is a reduction in transcription and a concomitant decrease in the levels of their primary transcripts. To test this, levels of miR-34a-5p and miR-132-3p pri-miRNA were investigated using individual TaqMan assays targeting the single-stranded region adjacent to the mature miRNA-containing stem loop. Using this approach, we found no significant change in pri-miR-34a expression at 20 min (with and without CPP), 5 h, or 24 h post-DBS, following normalization to HPRT (**Figure 4A**). These results suggest that a solely post-transcriptional mechanism regulates the levels of mature miR-34a-5p. In contrast, we found that the primary transcript of miR-132-3p was dramatically up-regulated 20 min post-DBS (*p* = 0.04, *n* = 5; **Figure 4B**). This increase was attenuated at 5 h (*p* = 0.33, *n* = 5; **Figure 4B**) and followed by a significant down-regulation at 24 h (*p* = 0.02, *n* = 4; **Figure 4B**). When DBS was delivered in the presence of CPP, the increase in the pri-miR-132 levels at 20 min was attenuated and was not significantly different from the LTP-stimulated, non-drug treated group (*p* = 0.08, *n* = 4; **Figure 4B**). This suggests that NMDAR activity was partially responsible for the LTP-related increase in miR-132-3p levels. Together these data show a striking discrepancy in the LTP regulation of the primary and mature transcripts of miR-132-3p and miR-212-3p, suggesting that the LTP-induced reduction in mature miR-132-3p levels was also regulated by post-transcriptional mechanisms.

### PREDICTED miR-34a-5p AND miR-132-3p TARGETS

Having found that miR-34a-5p and miR-132-3p were rapidly down-regulated following LTP induction, we set out to explore the biological significance of this by identifying potential targets for miR-34a-5p and miR-132-3p.Our initial approach was to investigate previously validated targets of these miRNA using Tarbase v6.0, a manually curated database comprising only experimentally validated miRNA:mRNA interactions (Vergoulis et al., 2012). This approach identified several plasticity-related genes as likely targets of miR-34a-5p and miR-132-3p (**Table 2**). These include *Arc*, and the glutamate receptor subunits *Grm7*, *Gria1, Grin2a, and Grin2b*; the latter two of which are rapidly up-regulated at synapses during LTP *in vivo* in a protein synthesis-dependent manner (Williams et al., 2007). A comparison of these validated targets with our previously published LTP-regulated mRNA data derived at 20 min (Ryan et al., 2011, 2012) highlighted *Arc,* and the calcium-dependent protease *Capn8,* both of which are up-regulated and targeted by miR-34a-5p. This analysis therefore supports the hypothesis that these miRNA play an important role in the consolidation and maintenance of LTP persistence by regulating the expression of specific plasticity-associated mRNA.

**Table 2:** Experimentally validated miRNA-target interactions for miR-34a-5p and miR-132-3p.

MiRNA	Gene symbol	Gene name	Reference	Prediction algorithm
miR-34a-5p	*Arc*	Activity related cytoskeletal protein	Wibrand et al. (2012)	miRanda
	*Capn8*	Calpain 8	Jeyaseelan et al. (2008)	miRanda, miRDB
	*E2f3*	E2F transcription factor 3	Pogribny et al. (2009)	PicTar, TargetScan
	*Grm7*	Glutamate receptor, metabotropic 7	Zhou et al. (2009)	PicTar, TargetScan
	*Mycn*	V-myc myelocytomatosis viral related oncogene, neuroblastoma derived (avian)	Pogribny et al. (2009)	miRanda, TargetScan, miRDB, miRWalk
	*Notch1*	Notch 1	Tryndyak et al. (2009)	PicTar, TargetScan, miRDB
	*Sirt1*	Sirtuin 1	Pogribny et al. (2009)	PicTar
	*Stx-1a*	Syntaxin 1A	Agostini et al. (2011)	
	*Syn1*	Synapsin I	Agostini et al. (2011)	
	*Syt1*	Synaptotagmin I	Agostini et al. (2011)	
	*Tagln*	Transgelin	Jeyaseelan et al. (2008)	
miR-132-3p	*Capn8*	Calpain 8	Jeyaseelan et al. (2008)	miRanda
	*Gria1*	Glutamate receptor, ionotropic, AMPA 1	Kawashima et al. (2010)	
	*Mecp2*	Methyl CpG binding protein 2 (Rett syndrome)	Klein et al. (2007); Mann et al. (2010)	PicTar, TargetScan
	*Mmp9*	Matrix metallopeptidase 9 (gelatinase B, 92kDa gelatinase, 92kDa type IV collagenase)	Jeyaseelan et al. (2008)	miRanda
	*Grin2a*	Glutamate receptor, ionotropic, *N*-methyl D-aspartate 2A	Kawashima et al. (2010)	
	*Grin2b*	Glutamate receptor, ionotropic, *N*-methyl D-aspartate 2B	Kawashima et al. (2010)	
	*p250GAP*	Rho GTPase activating protein 32	Vo et al. (2005)	PicTar

Focusing on miR-132-3p, we next extended our analyses to identification of novel targets. We undertook two approaches using target prediction algorithms developed to interrogate the transcriptome for potential binding sites for miRNA based on a number of characteristics, including sequence, thermodynamics, and conservation. Firstly, miRNA target predictions were obtained using the miRNA2function tool in the miRNA Body Map program (Mestdagh et al., 2011) and filtered to those predicted by at least three algorithms and those known to be expressed in rat CA1 neuropil (Cajigas et al., 2012). Using the rat CA1 neuropil data as an estimation of the transcripts present in the dentate gyrus neuropil, this analysis reduced the predicted 1,379 targets to four: *Ep300, Hn1, Klhl11,* and *Ptbp2*, of which *Ep300* and *Ptbp2* had previously been shown to interact with miR-132-3p (Alvarez-Saavedra et al., 2011; Smith et al., 2011). Our second approach involved using nine target prediction algorithms, selecting those targets predicted by at least four algorithms with sites in the rat genome and filtering according to the LTP KEGG pathway. This narrowed down the list of 3,965 putative targets to three: *Ep300, Gria2,* and *Mapk1*. These analyses therefore predicted that *Gria2, Hn1, Klhl11*, and *Mapk1* are novel targets of miR-132-3p.

### VALIDATION OF miR-132-3p TARGETS USING LUCIFERASE ASSAYS

**FIGURE 5:**
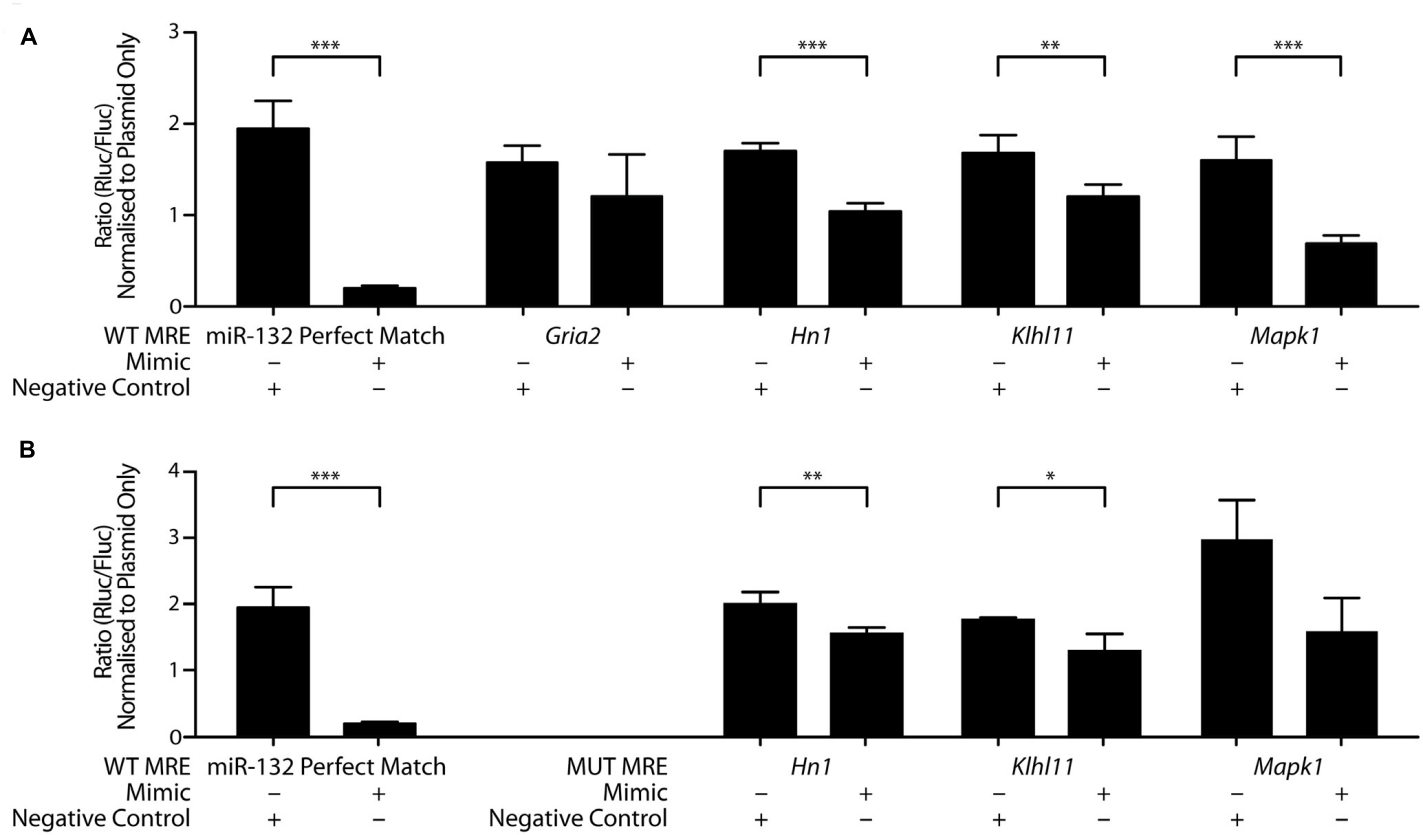
**MiR-132-3p binds to wild-type and mutant MRE of target gene transcripts.** Interactions between miR-132-3p and **(A)** four wild-type target MREs, and positive control and **(B)** miR-132-3p and three mutant target MREs, and positive control, as determined by dual luciferase assays. Significant down-regulation of the luminescence ratio with the miR-132-3p mimic when compared to the negative control was observed for wild-type *Hn1*, *Klhl11*, and *Mapk1*. *Gria2* showed no significant regulation. With mutant sequences, significant down-regulation of the luminescence ratio with the miR-132-3p mimic when compared to the negative control was observed for mutant *Hn1*, and *Klhl11*. Mutant *Mapk1* showed no significant regulation. Normalized to ratio of plasmid only. Average ratio ± SD, *n* = 4, independent two-tailed *t*-test, *p < 0.05, **p < 0.01, ***p < 0.001.

To test whether miR-132-3p can bind to these putative target gene transcripts, we carried out dual luciferase assays using the ψCheck-2 plasmid containing synthetic insert sequences (∼60 nucleotides) including the MREs and adjacent sequences from each mRNA 3′UTR (**Table 1**). This strategy was chosen to be able to detect any potential regulation that may be occluded from the use of the full 3′UTR (Cristino et al., 2014). Plasmids containing MREs for glutamate receptor, ionotropic, AMPA 2 (*Gria2),* hematological and neurological expressed 1 (*Hn1*), kelch-like family member 11 (*Klhl11*), mitogen-activated protein kinase 1 (*Mapk1*), or a perfect complementary sequence to miR-132-3p were co-transfected into COS-7 cells with miRIDIAN rno-miR-132-3p mimic. With *Renilla* luciferase expression under the control of the MRE following insertion, binding of the rno-miR-132-3p mimic to the MRE was measured as a decrease in *Renilla* luciferase activity as a result of decreased protein output. Co-transfection with the miR-132-3p perfect match sequence and the miR-132-3p mimic confirmed the efficacy of the assay: *Renilla* luciferase activity was significantly decreased in the presence of the miR-132-3p mimic compared to the negative control (**Figure 5A**). The same result was found for three of the four MRE inserts of interest (*Hn1*: *p* = 0.0002, *n* = 4; *Klhl11*: *p* = 0.007, *n* = 4; *Mapk1*: *p* < 0.0001, *n* = 4), indicating that miR-132-3p could regulate the expression of these three transcripts *in vivo*.

To test whether the seed site, an eight nucleotide long region located at the 3′ end of MRE considered important for the miRNA binding and function, plays a crucial role in mediating this binding, mutations within the MRE region for *Hn1*, *Klhl11*, and *Mapk1* were made by changing all guanines and cytosines to adenines (**Figure 5B**). Mutant *Hn1* (*p* = 0.05, *n* = 4) and *Klhl11* (*p* = 0.01, *n* = 4) MREs still significantly affected *Renilla* luciferase activity, suggesting that these seed sites are not critical and that 3′ compensatory binding is sufficient for regulation by miR-132-3p. In contrast, the mutated *Mapk1* MRE did not interact specifically with the miR-132-3p-3p mimic (*p* = 0.07, *n* = 4), suggesting that the seed site is necessary for miR-132-3p binding to *Mapk1*. Overall, these luciferase assays confirm *Hn1*, *Klhl11*, and *Mapk1* as novel targets for regulation by miR-132-3p.

### REGULATION OF p42-Mapk1 FOLLOWING LTP

**FIGURE 6:**
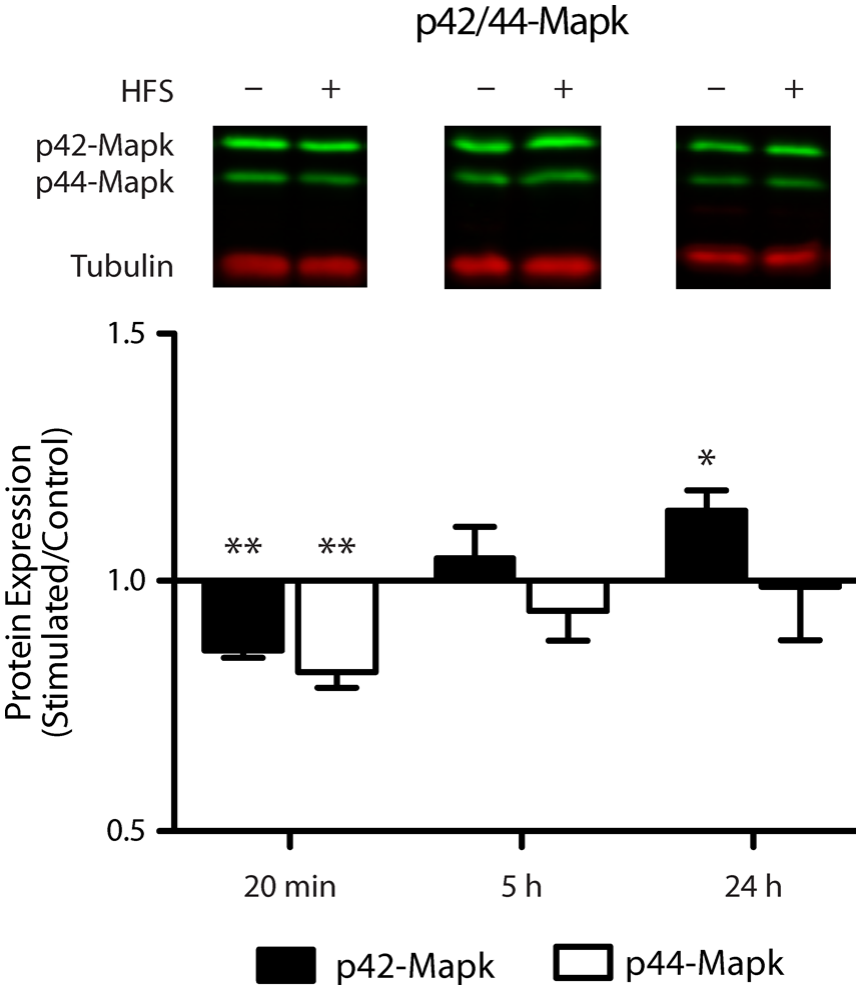
**Mapk1 protein levels are dynamically altered in response to LTP induction.** Western blot analysis showed a modest reduction in p42-Mapk1 levels at 20 min, which returned to baseline by 5 h and carried on to become an increase by 24 h post LTP induction. P42-Mapk3 levels were down-regulated at 20 min only. Data were normalized to tubulin and expressed relative to the matched contralateral control hemisphere (average ratio ± SEM, two-tailed *t*-test, *p < 0.05, **p < 0.01).

Given the prediction that *Mapk1* is a target of miR-132-3p and our observation that miR-132-3p is down-regulated 20 min following LTP, we hypothesized that this would be reflected in a subsequent up-regulation of Mapk1 protein levels. Using Western blot analysis of dorsal dentate gyrus extracts we found that the level of p42-Mapk1, the protein encoded by the *Mapk1* transcript, was indeed up-regulated but this change was not evident until 24 h following stimulation (*p* = 0.04, *n* = 4; **Figure 6**) and was preceded by a period of early down-regulation (20 min: *p* = 0.002, *n* = 4), which had recovered by 5 h (*p* = 0.51, *n* = 5). By contrast, there was no significant regulation of p44-Mapk, the protein encoded by the *Mapk3* transcript that does not have putative miR-132-3p target sites, at 5 h (*p* = 0.35) or 24 h (*p* = 0.91). However, like p42-Mapk1, we found a modest down-regulation at 20 min(*p* = 0.005).

## DISCUSSION

Here, we report a novel rapid down-regulation of a large cohort of miRNA following induction of LTP at perforant path synapses in adult awake freely moving animals. It is well established that the molecular events initiated following depolarisation do not solely contribute to the rapid post-translational events that potentiate synaptic transmission, but also activate processes to convert transient early phase LTP into its more persistent forms. These processes include regulated dendritic mRNA translation and new gene transcription (Abraham and Williams, 2003). Previous analyses have suggested that LTP-related gene expression is regulated first by activation of constitutively expressed transcription factors and that this response is then amplified through *de novo* expression of inducible transcription factors. However, as miRNA can coordinate the expression of numerous mRNA by their seed sites, miRNA-induced down-regulation may complement these transcriptional events, amplifying and possibly extending or diversifying the protein response following LTP induction. Our data suggest that down-regulation of miRNA, in particular miR-132-3p and miR-34a-5p, releases tonic inhibition and allows the expression of key plasticity-related proteins, including MAP kinase and glutamate receptor subunits which in turn may contribute to the consolidation of LTP. Furthermore, we provide evidence for the involvement of multiple mechanisms in the regulation of miRNA levels within neurons. Our findings differ from those reported by Wibrand et al. (2010, 2012) who, in anesthetized animals, found no rapid changes in miR-34a-5p or miR-132-3p, but an up-regulation of miR-132-3p at 2 h (Wibrand et al., 2010, 2012). As previous work has demonstrated that LTP-related genomic responses are curtailed in anesthetized rats (Jeffery et al., 1990), this suggests that the presence of anesthetic at the time of stimulation may also alter the LTP-related miRNA response.

### MECHANISM OF REGULATION OF microRNA LEVELS

Our findings of down-regulation of miRNA in response to plasticity induction are consistent with a previous report showing rapid decay of miRNA within retinal cells (Krol et al., 2010). By focusing on the plasticity-related miR-34a-5p (Agostini et al., 2011), we found that LTP results in a rapid and transient down-regulation of the mature transcript independent of any alteration in levels of its primary transcript. In contrast, we found a more succinct, transient down-regulation of miR-132-3p, with a concurrent rapid and transient increase in the levels of the miR-132/212 primary transcript, but no alteration in the co-transcribed mature miR-212-3p. This dichotomy suggests that there are specific post-transcriptional mechanisms regulating the expression of mature miRNA after LTP induction. In addition, our studies confirm a role for evoked NMDAR activation in the down-regulation of selected miRNA and demonstrate that this occurs at a post-transcriptional level.

While the regulation of miRNA levels post-LTP appear to be driven by neuronal activity mediated through the NMDAR receptor, the exact localization of these changes are unknown. Whole dentate gyri were used to profile the expression of miRNA. While this limits our ability to appreciate fully the effect of these changes in a localized manner, our findings of a generalized down-regulation in miRNA expression are consistent with our previous work showing increased mRNA expression (Ryan et al., 2011, 2012). Furthermore, the precise mechanisms underpinning the observed rapid down-regulation, including that of miR-132-3p and miR-34a-5p, are unknown. There are a number of possible post-transcriptional mechanisms that may underlie regulation of the mature miRNA, including modulation of miRNA stability via modification of 3′ terminal tail sequences. Indeed, the poly(A) polymerase Gld-2 has been linked not only to the regulation of miRNA stability, but also to memory and LTP persistence (Kwak et al., 2008; Katoh et al., 2009). It may be that these rapidly down-regulated miRNA lack these poly(A) tails, allowing them to be rapidly degraded, but later transcripts will be stabilized by Gld-2. Furthermore, Mapk1 has been shown to phosphorylate the trans-activation-responsive RNA-binding protein, Trbp, a Dicer accessory protein (Paroo et al., 2009), affecting the stability of the miRNA generating complex and the ability to form the RISC complex (Chendrimada et al., 2005). Thus, the pathways that lead to changes in miRNA stability already exist within the LTP gene networks and whether they are responsible for the rapid regulation of these miRNA is open for future research.

In striking contrast to the down-regulation of mature miR-132-3p transcript, the miR-132 primary transcript was found to be concurrently up-regulated at 20 min. What drives this increase in the miR-132/212 primary transcript? It seems likely that this involves the LTP-induced activation of CREB (Abraham et al., 2002) as the promoter region of pri-miR-132/212 is intergenic and contains a cAMP response element (CRE; Nudelman et al., 2010). The increase in levels of the primary transcript is likely constrained to the nucleus and may serve as a homeostatic mechanism, eventually being processed to replenish the pool of mature miRNA back to baseline by 5 h post-LTP induction. This mechanism may be employed to reset the levels of mature miR-132-3p and thereby contributing to an on-going capacity for long-term plasticity.

### FUNCTIONAL SIGNIFICANCE OF miR-34a-5p AND miR-132-3p DOWN-REGULATION

We have shown in an *in vitro* assay that the expression of *Mapk1* is likely to be regulated by miR-132-3p. Moreover, we have shown that there is dynamic regulation of Mapk1 protein levels following LTP. While further studies are required to substantiate the link between these two findings, they suggest that reduced miRNA has a delayed effect on Mapk1 protein levels. Our finding of a rapid down-regulation in Mapk1 levels contrasts with the reports of Wang et al. (2001) and Alzoubi and Alkadhi (2014), further emphasizing the effect that varied LTP induction protocols have on the molecular events elicited (Wang et al., 2001; Alzoubi and Alkadhi, 2014). Interestingly, previous studies have linked miR-132-3p and Mapk1, as levels of the pri-miR-132-3p/miR-212 cluster have been reported to be dependent on the Mapk pathway (Kawashima et al., 2010; Remenyi et al., 2010). Taken together with our new data, this predicts that a potentially homeostatic feedback loop exists between miR-132-3p and Mapk1 levels, a mechanism that is not uncommon in miRNA function (Tsang et al., 2007). Furthermore, as described above, Mapk1 can influence the stability of the Dicer complex, affecting the generation of new miRNA. As such, changing expression of miR-132-3p can have broader consequences beyond *Mapk1* regulation. This relationship may play an important role in the changes in gene expression post-LTP induction.

Very little is known about either *Hn1* or *Klhl11*, the other two genes that we identified as targets of miR-132-3p. Neither gene has been shown to be regulated following LTP induction at the mRNA level (Ryan et al., 2011, 2012). However, *Hn1* has previously been linked to regeneration of peripheral motor neurons and nervous system development (Zujovic et al., 2005). As remodeling of the neuron occurs during LTP, *Hn1* may be involved in this process. Even less is known about *Klhl11*; however, other members of the kelch-like family of genes are involved in a wide range of processes that are relevant to LTP, including inter/intracellular communication, cell morphology, cytoskeletal organization and protein binding (Dhanoa et al., 2013). These two novel targets of miR-132-3p offer interesting avenues for future research.

Further insight into the role of these miRNA in LTP can be gained by looking at other validated targets. Initiation of LTP requires activity-dependent release of glutamate, followed by postsynaptic events such as glutamate receptor trafficking and synthesis, extensive spine morphology modulation, altered gene expression, and neurotransmitter release modulation (Abraham and Williams, 2003). Our bioinformatic analysis predicts that regulation of translation by miRNA intersects with each of these key processes. For example, miR-34a-5p, which shares seed similarity to miR-34c-5p, elevated levels of which have been shown to be detrimental to memory (Zovoilis et al., 2011), has been shown to regulate the synthesis of *Arc*, a cytoskeletal protein involved in trafficking of AMPAR subunits (Wibrand et al., 2012), as well as *Grm7*, a metabotropic glutamate receptor subunit (Zhou et al., 2009), *Sirt1,* an epigenetic regulator of gene expression (Yamakuchi and Lowenstein, 2009), and the neurotransmitter release-related genes *Syt1, Syn1*, and *Stx-1a* (Agostini et al., 2011). Likewise, molecules confirmed to interact with miR-132-3p include *Mecp2*, a plasticity-related transcriptional repressor (Klein et al., 2007), *p250gap*, a brain-enriched GTPase-activating protein for Rho family GTPases involved in NMDAR-dependent actin reorganization (Wayman et al., 2008), and the activity-regulated glutamate receptor subunits *Gria1, Grin2a*, and *Grin2b* (Kawashima et al., 2010). Interestingly, we have previously shown that LTP induced in the dentate gyrus *in vivo* results in a rapid, protein synthesis-dependent, but transcription-independent, increase in the levels of NMDAR subunits found at synapses, which return to baseline within hours of the stimulation (Williams et al., 2003, 2007). As miR-132-3p has been reported to be located at synapses, these data suggest that synaptic levels of key glutamate receptor subunits are under the control of miR-132-3p. The exact mechanics of how this occurs, though, have yet to be determined.

Parallel to the investigation of whether regulation of these processes by miRNA contributes to LTP, there is a need to improve methods of identifying miRNA targets. One solution is to focus on targets that have been validated previously, but this pool of targets is very small, due to the lack of a high-throughput validation method. There is also potentially great value in the use of computational algorithms to identify putative targets in an unbiased manner. However, this approach is limited at present by a number of inherent challenges, in particular, how to narrow the large number of predicted targets, as the paucity of overlap between different algorithms often precludes the use of a consensus approach. In this study, one approach undertaken was to filter the predicted targets by dendritic mRNA, using CA1 neuropil data, given a lack of similar data for the dentate gyrus. It is acknowledged that this may exclude a population of dentate gyrus-specific transcripts and/or be misrepresentative of dendritic mRNA. The alternative approach of using annotation databases, such as the KEGG pathways, is also limited in its definition of genes involved in LTP. These limitations though do not detract from using these methods as a suitable starting point for study, as they use biological information to limit the extensive list of predicted targets. Nevertheless, this is an area that needs urgent development. Lastly, insight into which characteristics of miRNA-target binding are most important may provide improved methods of identifying targets, such as focusing on the thermodynamics of the interaction and the type of seed site. Such work may help to determine which target prediction tools are best to use.

## CONCLUSION

In conclusion, our data support the hypothesis that miRNA are important regulators of LTP-related gene expression. The rapid down-regulation of miRNA after LTP induction is a novel mechanism that may contribute to the expansive up-regulation of gene expression at this same time-point. This rapid down-regulation occurs via post-transcriptional mechanisms, at least for miR-34a-5p and miR-132-3p, and is likely to contribute to the consolidation of LTP at multiple levels, including amplifying the protein response. With these miRNA acting through crucial LTP genes like *Mapk1,* regulation of miRNA has the ability to affect the expression of a large number of genes. Therefore, the complexity of miRNA regulation during LTP consolidation clearly calls for future detailed studies to dissect the contributions and regulation of the individual miRNA involved.

## AUTHOR CONTRIBUTIONS

Greig Joilin: was the major contributor to the experimental aspects of the study. Greig Joilin contributed to the design of the study, isolated RNA, carried out RT-qPCRs, luciferase assays and bioinformatics experiments. Greig Joilin analyzed and interpreted the corresponding data and drafted the manuscript.

Diane Guévremont: isolated RNA and carried out the array study and the corresponding data analysis and critically revised the manuscript.

Brigid Ryan: contributed to the luciferase assays and bioinformatics experiments and critically revised the manuscript.

Charles Claudianos: design, interpretation and co-ordination of the luciferase study, critically revised the manuscript.

Alexandre S. Cristino: design and co-ordination of the luciferase study, undertook critical data analysis and interpretation.

Wickliffe C. Abraham: contributed to the design of the study, supervised the electrophysiology, data analysis and interpretation, and critically revised the manuscript.

Joanna M. Williams: conceived and participated in the design and co-ordination of the study, undertook data analysis and interpretation, and drafted and critically assessed the manuscript.

## Conflict of Interest Statement

The authors declare that the research was conducted in the absence of any commercial or financial relationships that could be construed as a potential conflict of interest.
